# Interrogating the bovine reproductive tract metagenomes using culture-independent approaches: a systematic review

**DOI:** 10.1186/s42523-021-00106-3

**Published:** 2021-06-09

**Authors:** Chian Teng Ong, Conny Turni, Patrick J. Blackall, Gry Boe-Hansen, Ben J. Hayes, Ala E. Tabor

**Affiliations:** 1 The University of Queensland, Queensland Alliance for Agriculture & Food Innovation, Centre for Animal Science, St Lucia, Brisbane, Queensland Australia; 2 The University of Queensland, School of Veterinary Science, Gatton, Queensland Australia; 3 The University of Queensland, School of Chemistry and Molecular Bioscience, St Lucia, Brisbane, Queensland Australia

**Keywords:** Microbiome, Reproductive, Cattle, Diversity, Fertility, Cow, Dysbiosis

## Abstract

Undesirable microbial infiltration into the female bovine reproductive tracts, for example during calving or mating, is likely to disturb the commensal microflora. Persistent establishment and overgrowth of certain pathogens induce reproductive diseases, render the female bovine reproductive tract unfavourable for pregnancy or can result in transmission to the foetus, leading to death and abortion or birth abnormalities. This review of culture-independent metagenomics studies revealed that normal microflora in the female bovine reproductive tract is reasonably consistently dominated by bacteria from the phyla Bacteroidetes, Firmicutes, Proteobacteria, following by Actinobacteria, Fusobacteria and Tenericutes. Reproductive disease development in the female bovine reproductive tract was demonstrated across multiple studies to be associated with high relative abundances of bacteria from the phyla Bacteroidetes and Fusobacteria. Reduced bacterial diversity in the reproductive tract microbiome in some studies of cows diagnosed with reproductive diseases also indicated an association between dysbiosis and bovine reproductive health. Nonetheless, the bovine genital tract microbiome remains underexplored, and this is especially true for the male genital tract. Future research should focus on the functional aspects of the bovine reproductive tract microbiomes, for example their contributions to cattle fertility and susceptibility towards reproductive diseases.

**Supplementary Information:**

The online version contains supplementary material available at 10.1186/s42523-021-00106-3.

## Introduction

In general, cattle reproduction (with a maximum of one pregnancy per year) is less efficient as compared to other livestock species which give birth to a litter or are oviparous [1–4].. On the herd level, the number of calves born and raised per breeding cycle is inevitably vital for economically sustainable dairy and beef production, as well as heifer replacements [5, 6]. Therefore, maintaining the bovine reproduction performance at an optimal level is a priority in cattle industries. Bovine reproduction performance is a multifactorial trait and can be affected by both infectious and non-infectious factors. Examples of non-infectious factors are genetic variation in fertility, environmental factors and nutrition [7, 8]. Infectious factors are primarily linked to persistent microbial colonization, which can lead to inflammation and compromised reproductive performances in various forms, including distorted reproductive cycle, reduced conception rate, increased risk of abortion, stillbirth and extended calving seasons [9–12].

During calving, microbes which are prevalent in livestock environments can gain access into the uterus of the cow [13, 14]. Typically, cattle can restore their uterus from postpartum microbial contamination within the first 5 weeks after calving, by uterus involution and discharge of the uterus and cervix content, as well as activation of host defence systems [15]. However, in some cases, bacteria can persistently colonize the reproductive tract and lead to inflammation [1, 16]. Aside from bacterial contamination during calving, other pathogens can gain access to the reproductive organs of the cow during mating and artificial insemination via contaminated sources [17–20]. Transmission of the causative agents from close contact with the contaminated environmental source or infected hosts potentially leads to bovine reproductive diseases, such as leptospirosis, neosporosis, brucellosis and bovine viral diarrhoea [21–24].

Establishment of opportunistic pathogens in the bovine reproductive tract reduced the cattle reproductive efficiency. Microbial infections in the cattle reproductive organs perturb the regulation of reproductive hormones. For example, in two separate studies using either cows with endometritis or those challenged with bacterial lipopolysaccharide (LPS), oestradiol levels were reduced and led to delayed ovulation [14, 25, 26]. In addition, it has been shown that bacterial LPS challenge disrupts the regulation of luteinizing hormone and prostaglandin F_2α_, lowering progesterone which negatively affects the viability of embryos by changing the uterine environment [27–29]. Another consequence of reduced levels of prostaglandin F_2α_ is the incomplete regression of the corpus luteum at the end of dioestrus, which led to low fertility [30–33]. Additionally, invasion by pathogenic microbes and their toxins induce inflammation, triggering host inflammatory responses and destruction of the endometrial integrity in cattle reproductive organs, which are unfavourable conditions for the transport of spermatozoa and embryonic development [34–36]. Infestation of pathogens on the bovine gametes detriments the reproductive efficiency by interfering the development of oocytes [37, 38] and reducing the sperm functionality including sperm viability, motility and DNA integrity [39–42]. Overgrowth of opportunistic pathogens is also not beneficial during early embryonic development as it increases the risk of early embryonic death, abortion or birth of an abnormal or persistently infected calf [43]. Bacterial species which were isolated from the bovine endometrial tissues using standard culture techniques include *Escherichia coli, Fusobacterium* spp., *Prevotella* spp. and *Trueperella pyogenes*. These bacteria have been hypothesized to be the causative pathogens responsible for postpartum endometrial pathology [4, 44, 45]. However, this hypothesis has been questioned extensively in recent years since the discovery of a previously underappreciated fraction of microbial composition using next generation metagenomics approaches [46–49].

The term metagenome refers to the collection of genomes and genes of the microorganisms from an environment [50]. The advent of DNA sequencing techniques allows decoding of both culturable and unculturable species concurrently and reveals the actual microbial community with high resolution [51–53]. In the area of host-pathogen interactions, the amount of information harvested efficiently using DNA sequencing approaches has been a breakthrough in deciphering the interplay between hosts and microbes [54]. Instead of focusing on the pathogenicity of a particular group of microbes, host-associated metagenomics studies unveil the role of the entire metagenome in determining host susceptibility to infectious diseases and the outcomes of infections [55]. Additionally, the significant role of the maternal reproductive tract microbiome associated with pregnancy outcomes and the subsequent early life of the progeny has been documented [56, 57]. To improve bovine reproductive performance, it would be beneficial to identify bovine reproductive tract microbiome biomarkers that can predict for high pregnancy chance or pregnancy risk.

Amplicon sequencing is a targeted sequencing approach focusing on a specific genomic region which are ubiquitous and discriminatory throughout the microbial population of interest [58, 59]. The common target genes used in amplicon sequencing are 16S rRNA genes for bacteria [60], 18S rRNA genes for eukaryotes [61] and internal transcribed spacer (ITS) genes for fungi [62]. The drawback of amplicon sequencing are the biases associated with the usage of different variable regions of the target genes as the amplicon primers [63]. The different binding affinities and resolution of each variable region across the taxa causes amplicon sequencing to selectively amplify certain reads and thus results in a distorted taxonomic prospect of the entire metagenome [64]. Additionally, amplicon sequencing renders limited resolution in functional profiling because it typically amplifies a small region of the target gene [65, 66]. Shotgun sequencing is a non-targeted approach in which all the genetic fragments in the sampled microbiome are sequenced [67, 68]. The individual sequence reads can be mapped directly to taxonomic databases or be assembled into contigs to provide more accurate information than is possible with other approaches. Reads and contigs generated from shotgun metagenomic studies can also be mapped to the protein and pathway databases for functional profiling or used for putative protein sequence identification [54, 69]. Shotgun sequencing provides a better resolution as compared to amplicon sequencing but at a higher cost and a greater data-processing effort. Hence, shotgun sequencing has been less widely used than amplicon sequencing for comparative metagenomics studies, which generally involve multiple samples [67, 68].

We have performed a systematic review to examine and summarise the available research articles on metagenomic sequencing studies of bovine reproductive tracts. The reported metagenome profiles of the bovine reproductive tracts were consolidated considering the rationale and study design in each study, in order to provide a systematic review of knowledge in this area.

## Materials and methods

### Search strategy and selection criteria

**Fig. 1:**
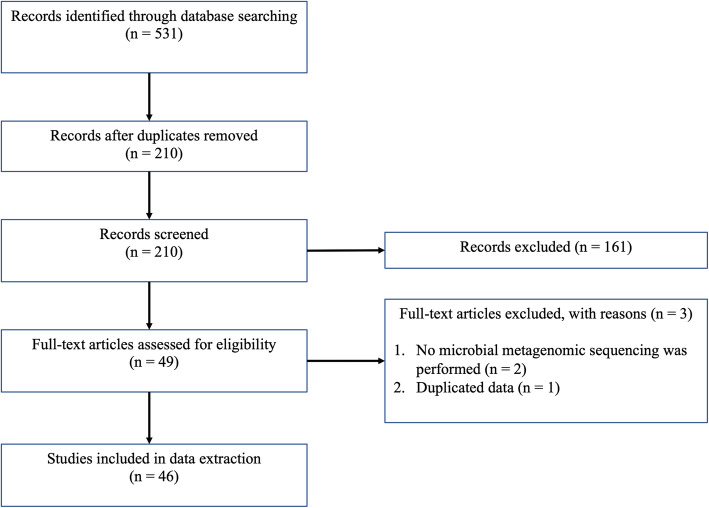
Flow chart of systematic review. The steps were adopted and modified from the Preferred Reporting Items for Systematic Reviews and Meta-Analyses (PRISMA) [71]

To assemble the available journal articles reporting metagenomics sequencing in bovine reproductive tracts, searches were conducted in six electronic databases (PubMed, Web of Science, Cochrane Library, Embase, Scopus and CABI) on 16th of September 2020. This systematic review was framed around the review title “metagenomics sequencing in bovine reproductive tract” by using the keywords “cattle”, “reproductive”, “metagenome” and “microbiome” for the searches. The detailed search strategies for each database are listed in Additional File 1. The records summoned by the databases were imported to EndNote [70] and the duplicates were removed. In this systematic review, records which did not report original data were excluded, including reviews, conference abstracts and book sections. The full-text articles were downloaded and articles which did not have English full text available were excluded. The title and abstracts of all records were screened to filter out studies which performed metagenomics analysis in hosts other than cattle, metagenome analysis in bovine organs other than the reproductive tract and non-metagenomics analyses. If the paper reported both culture-dependent and culture-independent, only culture independent results were taken into consideration. The steps of this systematic review were adopted and modified from the Preferred Reporting Items for Systematic Reviews and Meta-Analyses (PRISMA) [71] (Fig. 1).

### Data extraction and analysis

The included records were subjected to the next tier of screening to extract the following information: first author, journal name, year of publication, maturity and gender of the cattle, cattle breed, geographical location of the cattle, reproductive health condition, reproductive status, breeding method, sample size, specimen type, microbiome DNA isolation method, metagenomics sequencing platform, analysis platform, microbial phyla detected and rationale of the study. Data cleaning, stratification, analyses, and visualisation were performed in both Microsoft Excel [72] and R studio [73] with packages include dplyr [74], ggplot2 [75] and reshape2 [76].

### Stratification of bovine reproductive tract microbiome studies

The information extracted were used to categorise the included records. The geographical origins of the included studies were classified according to World Health Organisation (WHO) regional groupings [77], mainly European region, region of Americas and Western Pacific region. The sequencing platforms adopted in each of the included studies were stratified into three major categories: pyrosequencing, Illumina and Multiple/Other/Not specified. The sequencing regions in each study were stratified into 16S V1-V2, 16S V1-V3, 16S V3-V4, 16S V4, 16S V5-V6, shotgun and Multiple/Other/Not specified. Sample sizes were divided into less than 10, 11–50, 51–100, 101–200 and above 200. If samples were pooled together before sequencing, the number of pooled samples was used as the sample size instead of the number of samples. The category “Bovine reproductive tract” referred to the part of bovine reproductive tract from which the metagenomics sample was collected, mainly cow vagina and uterus. The specimen type collected from the bovine reproductive tract were categorised into biopsy, swab, wash or multiple if several types of specimens were collected. In general, biopsies were tissue samples incised from reproductive organs, swabs collected mucus using sterile tools such as cotton swabs, catheters or cytobrushes, while washes used saline injected into the reproductive organs to obtain a sample. DNA isolation methods were divided into five categories: column-based, magnetic-based, precipitation-based and Multiple/Other/Not specified. Column-based referred to DNA extraction depending on the solid-phase system in a spin-column for DNA purification; magnetic-based DNA extraction used magnetic beads for the separation of DNA; while precipitation-based DNA extraction utilised the isopropanol precipitation method as described previously [78].

The included studies were also stratified into different categories under “Rationale” according to the study objectives and primary findings, including baseline, breed, pathology, fertility and pregnancy stages, reproductive cyclicity stages, transmission and intervention studies. Baseline studies were designed to investigate the microbiome in cattle reproductive tract and did not include any comparative analysis. Studies which were stratified under the “breed” category compared the cattle reproductive tract microbiome between different breeds. Studies which were categorised under “pathology” compared the cattle reproductive tract microbiome between samples collected from healthy cattle and cattle with reproductive clinical diagnosis, including metritis, endometritis, purulent vaginal discharge and retained placenta. “Fertility/ Pregnancy stages” comprised of studies which compared the cattle reproductive tract microbiomes between non-pregnant and pregnant animals at different gestation stages. “Cyclicity stages” consisted of studies which determined the cattle reproductive tract microbiome at different reproductive cyclicity phases. Studies which examined the origin and the transmission of cattle reproductive tract microbiome were classified under “Transmission”. Studies which determined the effect of supplements, vaccines, antibiotics or treatments were categorised under “Intervention”.

## Results

**Fig. 2:**
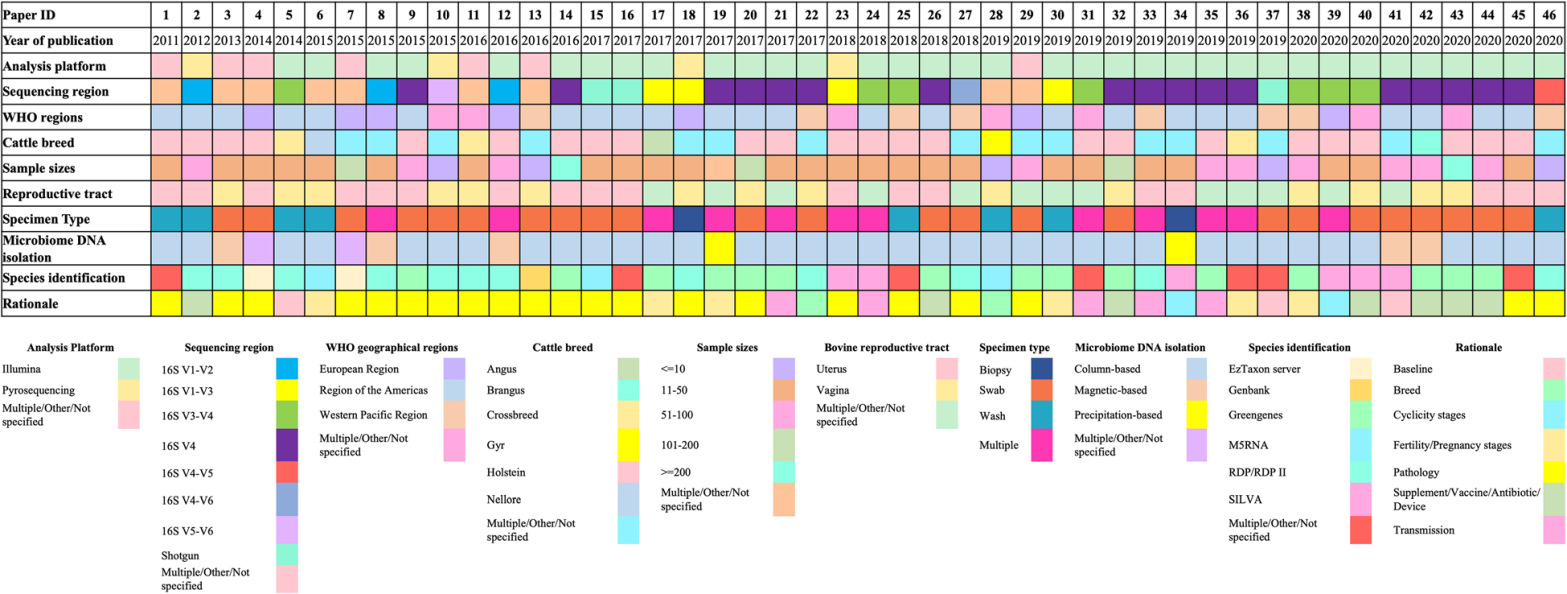
Overview of study design of 46 studies included in this systematic review. Each column depicts the study design of a paper which was represented with a paper identification number (ID) (Additional File 2). The rows describe the data extracted from the paper, including year of publication, sequencing platform, sequencing region, WHO geographical region, cattle breed, sample size, specimen type, microbiome DNA isolation method, taxonomy identification database and rationale. The colour codes for each data categories are listed in the Figure legend

In total, 531 records were retrieved from the customised searches. After excluding the duplicates, the first screening was conducted with 210 records. One hundred and sixty-two records were removed, including records which did not report original data (*n* = 58), records which do not have an English full text available (*n* = 11), studied animals were not cattle (*n* = 54), metagenome samples were not collected from bovine reproductive tract (n = 5) and non-metagenomics studies (*n* = 34). Full texts of the remaining records (*n* = 49) were assessed for eligibility and 46 records were eventually subjected to data extraction and stratification (Fig. 2).

**Fig. 3:**
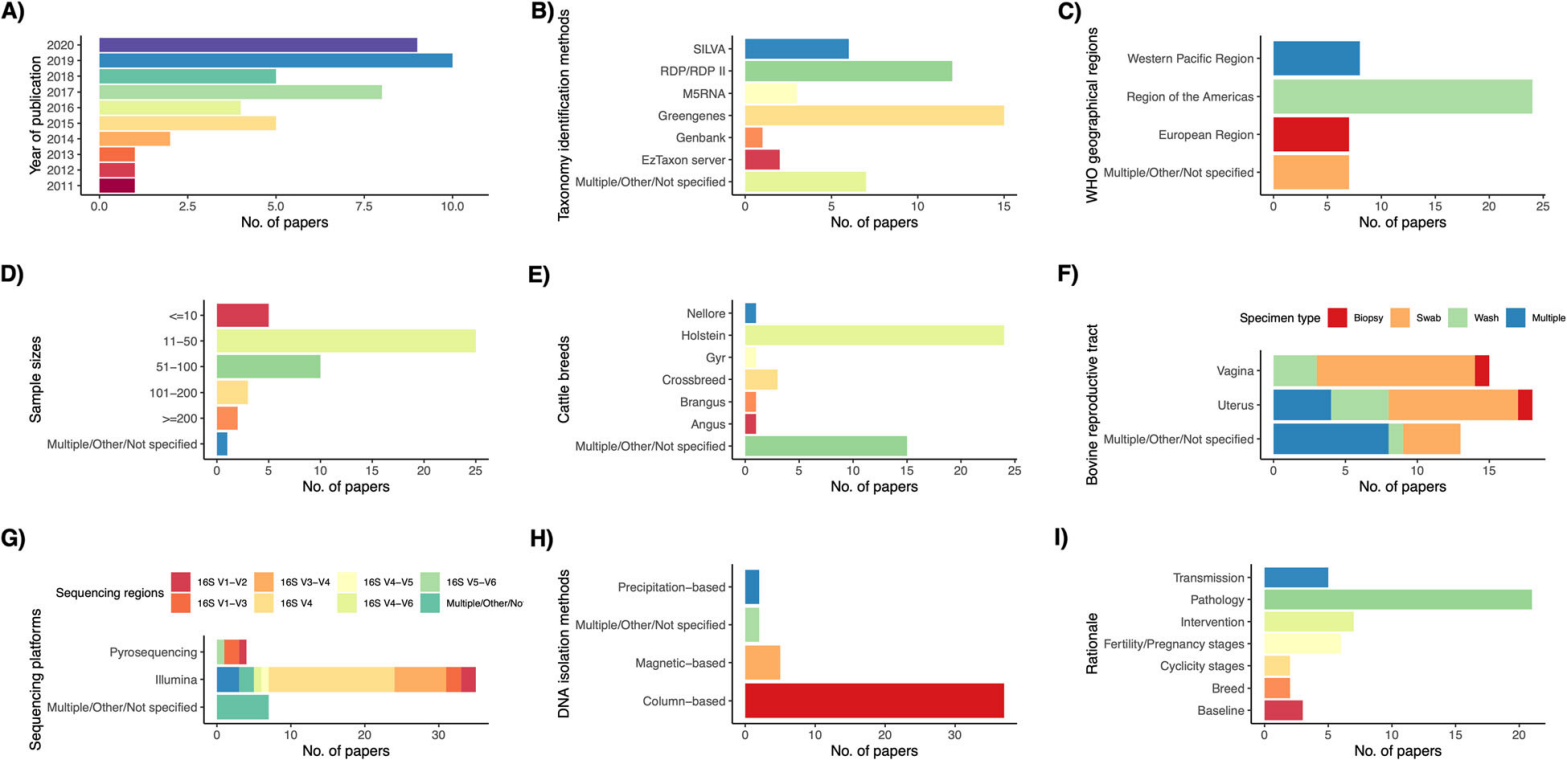
Number of papers stratified into different categories in **A**) year of publication, **B**) taxonomy identification method, **C**) WHO geographical region, **D**) sample size, **E**) cattle breed, **F**) specimen type, **G**) sequencing platform and sequencing region, **H**) microbiome DNA isolation method and I) rationale. Papers were classified as “Not specified” when the metadata was not available, “Multiple” if multiple study designs were implemented and “Other” if the study design was not commonly used

The bovine reproductive tract metagenomics papers included in this review were published between 2011 and 2020, with an increasing number of papers published in the 4 years from 2016 to 2020 (Fig. 3). Geographically, the studies reviewed in this paper were mainly from the Americas (*n* = 24), the Western Pacific region (*n* = 8), and Europe (*n* = 7) while the cattle breed which was most studied was the Holstein dairy breed. Twenty and sixteen studies selected for this review specifically targeted the metagenomes isolated from bovine uterus and vagina respectively, while the others investigated the metagenome from other or multiple parts of the bovine reproductive tract. Approximately 80% of the studies (*n* = 37) employed column-based DNA isolation methods to extract the genomic material from the microbiome samples while the precipitation-based, osmotic-based magnetic-based methods were less common. Illumina sequencing platform (~ 76%) was the most popular sequencing technology adopted by the studies to sequence bovine reproductive tract metagenomes, followed by Multiple/Other/Not specified (~ 15.2%) and pyrosequencing (8.7%). The majority of the studies included in this review focused on the bacterial metagenome by sequencing the 16S rRNA gene, particularly variable region V4. Assignment of taxonomic units in the studies included in this review were commonly performed using Greengenes (*n* = 15), followed by RDP/RDP II (*n* = 12) and SILVA (*n* = 6), while other studies conducted taxonomic unit identification using M5RNA, Genbank, EzTaxon server or multiple databases. Many of the studies (~ 43%) were conducted to examine the bovine reproductive metagenome of animals with reproductive disease, including metritis and endometritis. The bovine reproductive tract metagenome at different pregnancy stages and its causative relationship to cow fertility were explored by ~ 15% of the studies. Approximately 15% of the studies investigated the changes introduced to the bovine reproductive tract metagenome as the results of interventions of various supplements, vaccines, antibiotics and devices.

**Fig. 4:**
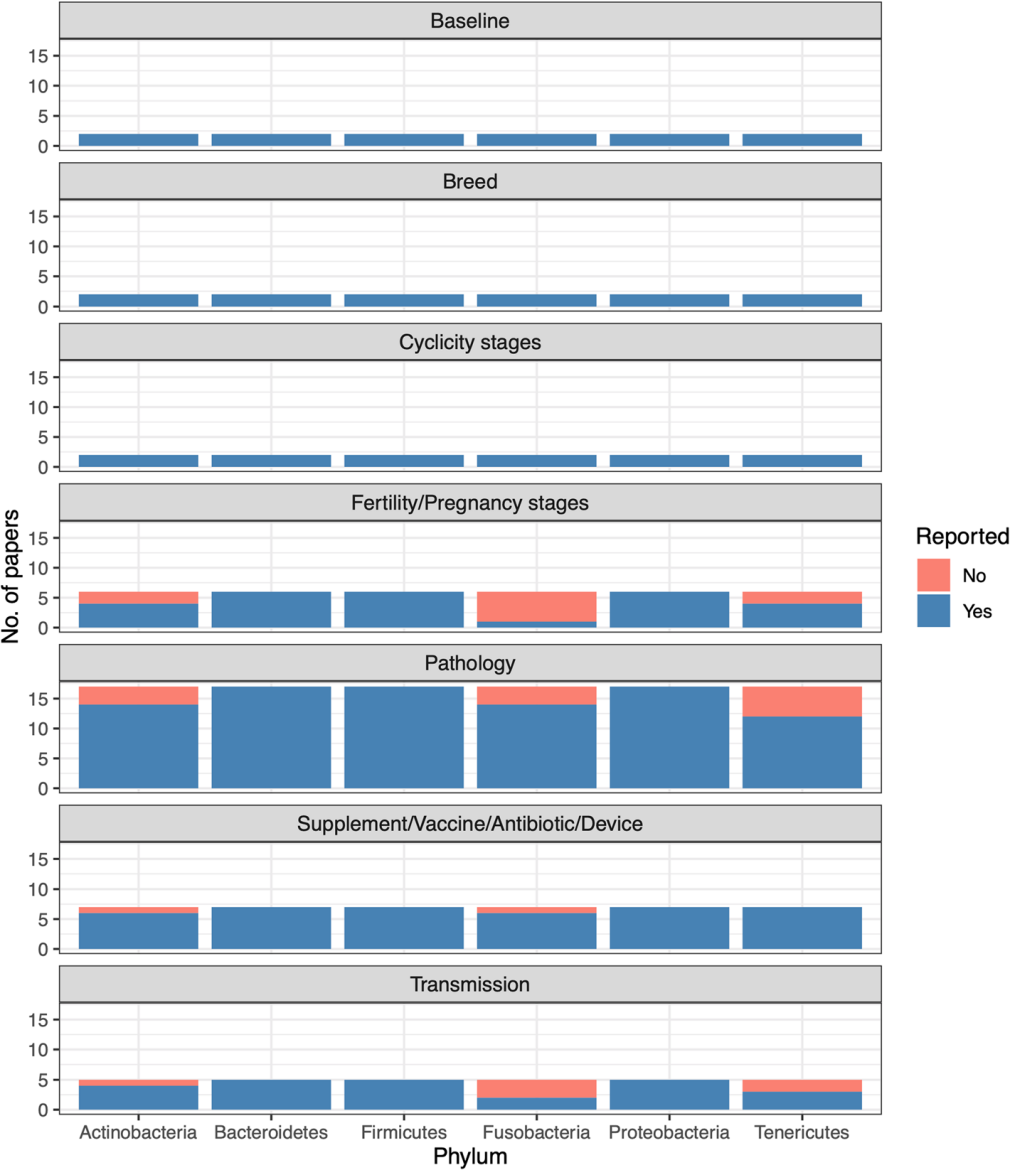
Number of papers reporting the six most abundant microbial phyla, including Actinobacteria, Bacteroidetes, Firmicutes, Fusobacteria, Proteobacteria and Tenericutes, under different study rationale. Blue represents the number of papers reported while red represents the number of papers did not report the respective bacterial phyla

Some of the papers were not included in the analysis of the most common bacterial taxonomic profile in bovine reproductive tract metagenomes because the paper did not report the common bacterial taxonomic profile (*n* = 2) or reported the common bacterial taxonomic profile generated using culture-dependent method (*n* = 3). The taxonomic profiles from various reports (*n* = 41) revealed that the most abundant bacterial phyla in the bovine reproductive tract were Bacteroidetes, Firmicutes and Proteobacteria, which were persistently identified in all studies, following by Actinobacteria, Fusobacteria and Tenericutes, which were detected in most of the studies (Fig. 4). Other microbial phyla which were reported in some of the studies reviewed include Spirochaetes, Verrucomicrobia, Lentisphaerae and Euryarchaeota.

## Discussion

### Culture-independent studies support existing understanding and provide new knowledge about bovine reproductive tract metagenomes

Bovine reproductive tract metagenomics studies reinforce our understanding of the microbial ecosystem within bovine reproductive systems. For example, the discovery of *Campylobacter fetus* subsp. *venerealis* in the metagenomics study investigating preputial samples from healthy bulls reinforced the previous findings regarding the role of bulls in spreading venereal diseases while remaining asymptomatic [17, 18, 20, 60]. Meanwhile, metagenomics studies also revealed that the lower reproductive tract of cows has a different microbial composition compared to primates and humans, which both have *Lactobacillus* spp. as the dominant genera in the vaginas of healthy individuals [79]. However, *Lactobacillus* spp. were detected in bovine vaginas at low levels, and this finding corresponds to the near-neutral pH in the bovine vagina, in sharp contrast to human vaginas [80, 81]. Additionally, metagenomics investigations disclosed that there were both shared and different core operational taxonomy units (OTUs) between the bovine vaginal and uterine samples. The shared OTUs indicated the interactions between the bacterial communities in the two reproductive organs while the different OTUs reiterated the differential microbial ecosystem niches as well as their attributes to the functional differences between the two reproductive organs [82]. The close resemblance of the vaginal microbiome and the microbiome in associated calves highlighted the possibility of vertical transmission of the maternal microbiome, which predetermines the health and survival rate of the calves [57, 83, 84]. A metagenomics study focused solely on the viruses also provided a baseline understanding of the genital tract virome of healthy dairy cattle [85].

High-throughput sequencing unveils a more detailed and accurate picture of the bovine genital tract microbiome. Intriguingly, some of the species, including *Bacillus* spp., *Enterococcus* spp., *Staphylococcus* spp. and *Streptococcus* spp., which were perceived as the normal microflora in the bovine genital tract via culture-based studies, were not significantly detected in the sequencing studies [86–88]. Similarly, *Escherichia* spp. and *Trueperella* spp., which were previously regarded as the causes of bovine metritis and endometritis by culture-dependent approaches, were reported with low abundances in reproductive disease-associated sequencing studies [89–93]. This observation is potentially attributed to the limitations of culturing in which the abundance of certain bacteria is masked selectively by enriched media and culturing conditions. However, it is also debatable if the main driver of bovine reproductive diseases is due to A) the high abundance of a specific bacterial species (or set of species) or B) the presence of key virulence factors associated with a minor population (which is more difficult to detect by metagenomics sequencing unless sequencing is very deep). Nevertheless, *Fusobacterium* spp. from the phylum Fusobacteria, *Prevotella* spp. (formerly *Bacteroides* spp.) and *Porphyromonas* spp. (formerly *Bacteroides* spp.) from the phylum Bacteroidetes were consistently identified to be associated with bovine reproductive diseases by both culture-dependant and culture-independent studies [49, 94, 95]. The studies highlighted the role of *Fusobacterium* spp., *Porphyromonas* spp. and *Prevotella* spp. in bovine reproductive infections. The mechanisms and the minimal infective concentrations of these species needs to be further examined to provide a better platform of knowledge for the development of diagnostic methods and treatments.

### Prevalence of microbial phyla in the bovine reproductive tract microbiome

Collectively, the bovine reproductive tract metagenomics studies have characterized the normal microflora at the phylum level in bovine reproductive organs. The commonly identified bacterial phyla in bovine reproductive tracts are Bacteroidetes, Firmicutes and Proteobacteria, followed by Actinobacteria, Fusobacteria and Tenericutes. Bacteria from these predominant phyla form the commensal microbiome in cattle reproductive organs, regardless of the breed, farm, gender, geographical location, sampling site, reproductive status and reproductive health. Notably, these bacterial phyla were also commonly detected in gastrointestinal tract microbial studies [96–98]. The close anatomical proximity between the two systems may have allowed the colonization of the reproductive tract microbial community originating from the gastrointestinal tract [56, 82, 99]. In addition, the possibility of direct faecal and environmental contamination of the reproductive organs should be taken into consideration [60, 82].

Interestingly, there was no exclusive association of a specific bacterial phyla with bovine reproductive diseases consistently reported across the studies. Several studies reported that the reproductive organs of both diseased and healthy cows shared the same dominant bacterial phyla, including Bacteroidetes, Firmicutes, Fusobacteria and Proteobacteria, but at different proportions [48, 94, 100–103]. Proteobacteria and Firmicutes were dominant in healthy cows while Fusobacteria and Bacteroidetes were prevalent in the reproductive tract of cows that eventually developed reproductive disease after parturition. Similar results were also derived from two shotgun sequencing studies, one that investigated the uterine microbiome of cows diagnosed with metritis [104] and one that investigated the uterine microbiome of cows with purulent vaginal discharge [105]. In both studies, the same sets of bacterial phyla were present in the uterus of both healthy cows and cows with reproductive diseases, but with increased abundance of Fusobacteria and Bacteroidetes in diseased animals.

It was observed that the low Firmicutes to Bacteroidetes ratio was an early sign in cows who subsequently develop postpartum endometritis [106]. The significance of the presence of Firmicute lactic acid bacteria, particularly *Lactobacillus* spp., in maintaining the vaginal homeostasis has been well established [107]. *Lactobacillus* spp. are not the dominant Firmicutes in bovine reproductive tracts, however the dominance of other lactic acid bacteria, such as *Enterococcus* spp. and *Streptococcus* spp., has been identified in bovine reproductive tracts [86, 108]. Lactic acid bacteria convert glycogen into lactic acid, creating an environment with a low pH level that inhibits the growth of pathogenic microbes [109]. Additionally, lactic acid bacteria also exert antimicrobial effects by producing compounds, such as bacteriocins, defensins and hydrogen peroxide, to facilitate their survival over other bacteria [110, 111]. Overgrowth of opportunistic bacteria from the phyla Bacteroidetes and Fusobacteria at the expense of Firmicutes contributes to the development of bovine reproductive diseases. A recent culture-independent investigation with droplet digital PCR confirmed that *Prevotella melaninogenica* (Bacteroidetes) and *Fusobacterium necrophorum* (Fusobacteria) were the causative agents responsible for metritis in cattle [112]. Several studies have suggested a strong synergetic interaction between Bacteroidetes and Fusobacteria causes bovine metritis and endometritis [36, 94, 113]. *Fusobacterium* spp. stimulate Bacteroidetes proliferation by providing growth factors and by releasing a toxic protein against the host leukocytes to weaken the host defence [114, 115]. Hence, *Prevotella melaninogenica*, which possess collagenolytic activity, can disrupt the epithelial integrity even when present in low numbers [116].

### Biodiversity in bovine reproductive tract microbiome

Microbial biodiversity is governed by two major factors, which are the number of species present in the community (richness) and the relative abundance of each species (evenness) [117]. Dysbiosis, which is the shift of commensal communities, [118, 119], was observed in some metagenomics studies comparing different pregnancy status, reproductive health conditions and fertility.

Metagenomic-based investigations of the bovine genital tract illustrated the potential association between the shifts of microbial abundancies and the hormonal changes within the bovine reproductive system during different stages of the reproductive cycle. In an experiment characterizing the vaginal microbiome of heifers, non-pregnant cows, primiparous and multiparous cows, a lower bacterial composition and a higher archaeal abundance were observed in the pregnant cows [99]. Since progesterone is the dominant hormone during pregnancy, the level of progesterone hormone and the microbial community structure are likely to be inter-dependent. On the other hand, the shifts of bacterial diversity in the uterus during the oestrus synchronization program was also observed, reinforcing the effects of hormonal changes on the bacterial composition [120]. This finding coincides with the shifts of vaginal microbial communities during the oestrous cycle as illustrated previously in a culture-dependent analysis, in which a decrease in the abundance of aerobes and facultative anaerobes was observed when progesterone levels are high [86]. A rise in estrogen levels stimulates the production and accumulation of glycogen while high progesterone levels suppress glycogen synthesis [121–123]. Depolymerization of glycogen, either by the host or amylase-producing microbes, releases nutrients for the microbial communities in the reproductive tract [124]. Hence the reduced numbers of microbes and consequently the reduced biodiversity are attributed to low levels of estrogen and high levels of progesterone, as occurs for example during luteal phase and pregnancy.

The association between dysbiosis and bovine reproductive infections was observed in some of the metagenomic studies. Low microbial diversity was also the differentiating factor which separated vaginal microbiome samples isolated from cows that developed bovine necrotic vulvovaginitis from those that developed mild vulvovaginitis [125]. Multiple metagenomics studies reported lower levels of microbial diversity in the reproductive tract samples from cows with reproductive diseases, which contrasted with the complex bacterial communities sampled from healthy cows [95, 126–132]. These studies suggested that the opportunistic bacteria from phyla Bacteroidetes and Fusobacteria overgrow other members in the microflora, resulting in a disturbed microbiome with low microbial diversity. However, the occurrence of bacterial dysbiosis and an association with reproductive disease development and low fertility were observational and not observed in some disease-associated metagenomics studies (Additional File 3) [101, 104, 105]. Therefore, it is questionable whether the low microbial diversity can be adopted as an indicator for pathology. Further investigations need to be conducted to examine the inconsistent findings, by ruling out the different biases introduced by experimental designs and sequencing strategies such as sequencing depth.

### Functional aspects of bovine reproductive tract microbiome

The growing interest of sequencing microbiome samples using shotgun approaches is attributed to a benefit in characterizing the functional profiles with the information encoded within the contigs. Functional analysis revealed that the uterine microbiome of cows with metritis expressed a significantly higher amount of genes coding for “protein transportation across cytoplasmic membrane” and “type IV bacterial secretion systems” [104]. Secretion and invasion of virulence factors into host cells via the cellular membranes are common pathological mechanisms [133, 134], and are conjectured to aid in the colonization of uterine epithelial cells and the invasion of the mucosal surface by bovine metritis-causing bacteria [135]. Genes transcribing for “LPS modification” and “cytolethal distending toxins (CDTs)” have been exclusively and highly expressed in the uterine microbiome of cows diagnosed with purulent vaginal discharge [105]. The highly variable LPS modification systems of invading Gram-negative bacteria increase their survival opportunities in hosts, e.g. by escaping the host antimicrobial defence mechanisms, subsequently leading to persistent and chronic infections [136, 137]. Gram-negative bacteria also secrete CDTs to trigger G2/M cell cycle arrest and cause apoptosis by inducing the enlargement of the mammalian cells [138]. Examples of CDT coding bacterial species are *Escherichia coli, Campylobacter* spp. and enterohepatic *Helicobacter* spp. [139]. Furthermore, the exclusive expression of genes coding for tolerance to colicin E2 in the microbiome of healthy cows is intriguing [104, 105]. Colicin E2 is an antibiotic nuclease which exhibits inhibitory effects by binding onto the outer membrane receptors of targeted bacteria [140]. The tolerance to colicin E2 posed by the uterine microbiome of healthy cows represses the overgrowth of harmful pathogens [104, 105].

### Future prospects

The bovine reproductive tract microbiome is relatively underexplored, particularly in terms of specific taxonomic classification and functional aspect of the microbiome, which are beneficial for the development of diagnostic methods, such as microbial biomarkers and dysbiosis indexes. Further investigations are essential to provide more meaningful and supportive information to aid development of novel treatment for bovine infertility and reproductive illness, based on altering the microbiome of the reproductive tract, such as microbiome transplantation.

## Conclusions

Despite the different experimental designs and approaches, the bovine reproductive tract microbiome studies reported the most common bacterial phyla in bovine reproductive organs are Bacteroidetes, Proteobacteria and Firmicutes, followed by Fusobacteria, Actinobacteria and Tenericutes. The shift of microbial composition, with elevated abundancies of bacterial phyla Bacteroidetes and Fusobacteria in the reproductive tract metagenomes from cows with reproductive diseases, emphasized a pivotal relationship of the members from these two bacterial phyla with bovine reproductive disease development. Further analyses are needed to examine whether the shifts in microbial community compositions are the reason for higher susceptibility of the animals towards reproductive diseases or the result of the reproductive disease. Dysbiosis was observed in some studies that investigated the metagenomes of healthy cattle and cattle diagnosed with reproductive diseases. It is crucial to determine whether the low microbial diversity is truly representative of a disease process or a distorted view of the bovine reproductive tract microbiome due to inherent biases of the experimental design and sequencing methodologies.

## Supplementary Information

**Additional file 1.** Keywords and search strategies for each database.

**Additional file 2.** Data extraction and stratification from 46 papers included in systematic review.

**Additional file 3.** Biodiversity indexes from 46 papers included in systematic review.

## Data Availability

Not applicable.

## Abbreviations

LPS: Lipopolysaccharides
ORF: Open reading frame
OTU: Operational taxonomy units
