# Supplementary material for: Interrogating the bovine reproductive tract metagenomes using culture-independent approaches: a systematic review
Source: Anim Microbiome. 2021 Jun 9;3:41. doi: 10.1186/s42523-021-00106-3 (PMC8191003; doi:10.1186/s42523-021-00106-3)
Supplement: Supplementary file 1 — Additional file 1. Keywords and search strategies for each database. [file 42523_2021_106_MOESM1_ESM.pdf]

Additional File 1: Keywords and search strategies for each database

Pubmed (Title and abstract):
((genital[Title/Abstract] OR reproductive[Title/Abstract] OR uterine[Title/Abstract] OR uterus[Title/Abstract] OR vaginal[Title/Abstract] OR vagina[Title/Abstract] OR vaginas[Title/Abstract] OR cervical[Title/Abstract] OR cervix[Title/Abstract] OR preputial[Title/Abstract] OR prepuce[Title/Abstract] OR semen[Title/Abstract]) AND (cattle[Title/Abstract] OR cow[Title/Abstract] OR cows[Title/Abstract] OR bull[Title/Abstract] OR bulls[Title/Abstract] OR bovine[Title/Abstract] OR heifer[Title/Abstract] OR heifers[Title/Abstract] OR beef[Title/Abstract])) AND (metagenome[Title/Abstract] OR metagenomic[Title/Abstract] OR metagenomics[Title/Abstract] OR microbiome[Title/Abstract] OR microbiomes[Title/Abstract] OR microbiota[Title/Abstract] OR high-throughput sequencing[Title/Abstract])

Embase (Title, abstract and keyword)
(genital:ti,ab,kw OR reproductive:ti,ab,kw OR uterine:ti,ab,kw OR uterus:ti,ab,kw OR vaginal:ti,ab,kw OR vagina:ti,ab,kw OR vaginas:ti,ab,kw OR cervical:ti,ab,kw OR cervix:ti,ab,kw OR preputial:ti,ab,kw OR prepuce:ti,ab,kw OR semen:ti,ab,kw) AND (cattle:ti,ab,kw OR cow:ti,ab,kw OR cows:ti,ab,kw OR bull:ti,ab,kw OR bulls:ti,ab,kw OR bovine:ti,ab,kw OR heifer:ti,ab,kw OR heifers:ti,ab,kw OR beef:ti,ab,kw) AND (metagenome:ti,ab,kw OR metagenomic:ti,ab,kw OR metagenomics:ti,ab,kw OR microbiome:ti,ab,kw OR microbiomes:ti,ab,kw OR microbiota:ti,ab,kw OR 'high-throughput sequencing':ti,ab,kw)

Cochrane library (Title, abstract and keyword)
genital OR reproductive OR uterine OR uterus OR vaginal OR vagina OR vaginas OR cervical OR cervix OR preputial OR prepuce OR semen in Title Abstract Keyword AND cattle OR cow OR cows OR bull OR bulls OR bovine OR heifer OR heifers OR beef in Title Abstract Keyword AND metagenome OR metagenomic OR metagenomics OR microbiome OR microbiomes OR microbiota OR high-throughput sequencing in Title Abstract Keyword - (Word variations have been searched)

Web of Science Core Collection (Topic)
TOPIC: (genital OR reproductive OR uterine OR uterus OR vaginal OR vagina OR vagi nas OR cervical OR cervix OR preputial OR prepuce OR semen) *AND* **TOPIC:** (cattle O R cow OR cows OR bull OR bulls OR bovine OR heifer OR heifers OR beef) *AND* **TOP IC:** (metagenome OR metagenomic OR metagenomics OR microbiome OR microbiomes OR microbiota OR high-throughput sequencing)

Scopus (Title, abstract and keyword)
(TITLE-ABS-KEY (genital OR reproductive OR uterine OR uterus OR vaginal OR vagina OR vaginas OR cervical OR cervix OR preputial OR prepuce OR semen) AND TITLE-ABS-KEY (cattle OR cow OR cows OR bull OR bulls OR bovine OR heifer OR heifers OR beef) AND TITLE-ABS-KEY (metagenome OR metagenomic OR metagenomics OR microbiome OR microbiomes OR microbiota OR "high-throughput sequencing"))

CABI (all fields)
(genital OR reproductive OR uterine OR uterus OR vaginal OR vagina OR vaginas OR cervical OR cervix OR preputial OR prepuce OR semen) AND (cattle OR cow OR cows OR bull OR bulls OR bovine OR heifer OR heifers OR beef) AND (metagenome OR metagenomic OR metagenomics OR microbiome OR microbiomes OR microbiota OR "high-throughput sequencing")
