# Supplementary material for: Interrogating the bovine reproductive tract metagenomes using culture-independent approaches: a systematic review
Source: Anim Microbiome. 2021 Jun 9;3:41. doi: 10.1186/s42523-021-00106-3 (PMC8191003; doi:10.1186/s42523-021-00106-3)
Supplement: Supplementary file 2 — Additional file 2. Data extraction and stratification from 46 papers included in systematic review. [file 42523_2021_106_MOESM2_ESM.pdf]

Additional File 2: Data extraction and stratification from 46 papers included in systematic review.

Paper ID	Year of Publication	Analysis Platform	Sequencing Region	WHO Regions	Cattle Breed	Sample Sizes	Reproductive tract	Specimen type	Microbiome DNA Isolation	Species Identification	Rationale
1	2011	Multiple/Other/Not specified	Multiple/Other/Not specified	Region of the Americas	Holstein	11-50	Uterus	Wash	Column-based	Multiple/Other/Not specified	Pathology
2	2012	Pyrosequencing	16S V1-V2	Region of the Americas	Holstein	51-100	Uterus	Wash	Column-based	RDP/RDP II	Intervention
3	2013	Multiple/Other/Not specified	Multiple/Other/Not specified	Region of the Americas	Holstein	11-50	Vagina	Swab	Magnetic-based	RDP/RDP II	Pathology
4	2014	Multiple/Other/Not specified	Multiple/Other/Not specified	European Region	Holstein	11-50	Uterus	Swab	Multiple/Other/Not specified	EzTaxon server	Pathology
5	2014	Illumina	16S V3-V4	Region of the Americas	Crossbreed	11-50	Vagina	Wash	Column-based	RDP/RDP II	Baseline
6	2015	Illumina	Multiple/Other/Not specified	Region of the Americas	Nellore	11-50	Vagina	Wash	Column-based	M5RNA	Fertility/Pregnancy stages
7	2015	Multiple/Other/Not specified	Multiple/Other/Not specified	European Region	Multiple/Other/Not specified	101-200	Uterus	Swab	Multiple/Other/Not specified	EzTaxon server	Pathology
8	2015	Illumina	16S V1-V2	European Region	Multiple/Other/Not specified	11-50	Uterus	Multiple	Magnetic-based	RDP/RDP II	Pathology
9	2015	Illumina	16S V4	Region of the Americas	Holstein	51-100	Uterus	Swab	Column-based	Greengenes	Pathology
10	2015	Pyrosequencing	16S V5-V6	Multiple/Other/Not specified	Multiple/Other/Not specified	<=10	Vagina	Swab	Column-based	RDP/RDP II	Pathology
11	2016	Multiple/Other/Not specified	Multiple/Other/Not specified	Multiple/Other/Not specified	Crossbreed	11-50	Vagina	Swab	Column-based	RDP/RDP II	Pathology
12	2016	Illumina	16S V1-V2	European Region	Holstein	51-100	Uterus	Multiple	Magnetic-based	RDP/RDP II	Pathology
13	2016	Multiple/Other/Not specified	Multiple/Other/Not specified	Western Pacific Region	Multiple/Other/Not specified	<=10	Vagina	Swab	Column-based	Genbank	Pathology
14	2016	Illumina	16S V4	Region of the Americas	Holstein	>=200	Uterus	Swab	Column-based	Greengenes	Pathology
15	2017	Illumina	Shotgun	Region of the Americas	Holstein	11-50	Uterus	Swab	Column-based	M5RNA	Pathology
16	2017	Illumina	Shotgun	Region of the Americas	Holstein	11-50	Uterus	Swab	Column-based	Multiple/Other/Not specified	Pathology
17	2017	Illumina	16S V1-V3	Region of the Americas	Angus	11-50	Multiple/Other/Not specified	Multiple	Column-based	Greengenes	Fertility/Pregnancy stages
18	2017	Pyrosequencing	16S V1-V3	European Region	Multiple/Other/Not specified	11-50	Vagina	Biopsy	Column-based	RDP/RDP II	Pathology
19	2017	Illumina	16S V4	Region of the Americas	Multiple/Other/Not specified	Multiple/Other/Not specified	Multiple/Other/Not specified	Multiple	Precipitation-based	Greengenes	Fertility/Pregnancy stages
20	2017	Illumina	16S V4	Region of the Americas	Holstein	101-200	Vagina	Swab	Column-based	Greengenes	Pathology
21	2017	Illumina	16S V4	Region of the Americas	Holstein	11-50	Multiple/Other/Not specified	Multiple	Column-based	Greengenes	Transmission
22	2017	Illumina	16S V4	Western Pacific Region	Multiple/Other/Not specified	11-50	Vagina	Swab	Column-based	RDP/RDP II	Breed
23	2018	Pyrosequencing	16S V1-V3	Multiple/Other/Not specified	Holstein	11-50	Uterus	Multiple	Column-based	SILVA	Pathology
24	2018	Illumina	16S V3-V4	Region of the Americas	Holstein	11-50	Multiple/Other/Not specified	Multiple	Column-based	SILVA	Transmission
25	2018	Illumina	16S V3-V4	Western Pacific Region	Holstein	11-50	Uterus	Wash	Column-based	Multiple/Other/Not specified	Pathology
26	2018	Illumina	16S V4	Region of the Americas	Holstein	11-50	Uterus	Swab	Column-based	Greengenes	Intervention

27	2018	Illumina	16S V4-V6	Western Pacific Region	Multiple/Other/Not specified	11-50	Multiple/Other/Not specified	Swab	Column-based	RDP/RDP II	Pathology
28	2019	Illumina	Multiple/Other/Not specified	Multiple/Other/Not specified	Gyr	<=10	Vagina	Wash	Column-based	M5RNA	Breed
29	2019	Multiple/Other/Not specified	Multiple/Other/Not specified	European Region	Multiple/Other/Not specified	51-100	Multiple/Other/Not specified	Swab	Column-based	Greengenes	Pathology
30	2019	Illumina	16S V1-V3	Region of the Americas	Multiple/Other/Not specified	11-50	Multiple/Other/Not specified	Wash	Column-based	Greengenes	Fertility/Pregnancy stages
31	2019	Illumina	16S V3-V4	Multiple/Other/Not specified	Holstein	11-50	Multiple/Other/Not specified	Multiple	Column-based	Multiple/Other/Not specified	Transmission
32	2019	Illumina	16S V4	Region of the Americas	Holstein	101-200	Vagina	Swab	Column-based	Greengenes	Intervention
33	2019	Illumina	16S V4	Western Pacific Region	Multiple/Other/Not specified	11-50	Uterus	Multiple	Column-based	RDP/RDP II	Transmission
34	2019	Illumina	16S V4	Region of the Americas	Multiple/Other/Not specified	11-50	Uterus	Biopsy	Precipitation-based	SILVA	Cyclicity stages
35	2019	Illumina	16S V4	Region of the Americas	Holstein	51-100	Multiple/Other/Not specified	Multiple	Column-based	Greengenes	Transmission
36	2019	Illumina	16S V4	Region of the Americas	Crossbreed	51-100	Multiple/Other/Not specified	Multiple	Column-based	Multiple/Other/Not specified	Fertility/Pregnancy stages
37	2019	Illumina	Shotgun	Western Pacific Region	Multiple/Other/Not specified	<=10	Multiple/Other/Not specified	Swab	Column-based	Multiple/Other/Not specified	Baseline
38	2020	Illumina	16S V3-V4	Western Pacific Region	Holstein	51-100	Vagina	Swab	Column-based	Greengenes	Fertility/Pregnancy stages
39	2020	Illumina	16S V3-V4	European Region	Holstein	11-50	Multiple/Other/Not specified	Multiple	Column-based	SILVA	Cyclicity stages
40	2020	Illumina	16S V3-V4	Multiple/Other/Not specified	Holstein	11-50	Vagina	Swab	Column-based	SILVA	Intervention
41	2020	Illumina	16S V4	Region of the Americas	Multiple/Other/Not specified	51-100	Multiple/Other/Not specified	Swab	Magnetic-based	SILVA	Baseline
42	2020	Illumina	16S V4	Region of the Americas	Brangus	51-100	Vagina	Swab	Magnetic-based	Greengenes	Intervention
43	2020	Illumina	16S V4	Multiple/Other/Not specified	Holstein	>=200	Vagina	Swab	Column-based	Greengenes	Intervention
44	2020	Illumina	16S V4	Region of the Americas	Holstein	51-100	Uterus	Swab	Column-based	Greengenes	Intervention
45	2020	Illumina	16S V4	Region of the Americas	Holstein	11-50	Uterus	Swab	Column-based	Multiple/Other/Not specified	Pathology
46	2020	Illumina	16S V4-V5	Western Pacific Region	Multiple/Other/Not specified	<=10	Uterus	Wash	Column-based	RDP/RDP II	Pathology
