# Supplementary material for: Interrogating the bovine reproductive tract metagenomes using culture-independent approaches: a systematic review
Source: Anim Microbiome. 2021 Jun 9;3:41. doi: 10.1186/s42523-021-00106-3 (PMC8191003; doi:10.1186/s42523-021-00106-3)
Supplement: Supplementary file 3 — Additional file 3. Biodiversity indexes from 46 papers included in systematic review. [file 42523_2021_106_MOESM3_ESM.pdf]

Additional File 3: Biodiversity indexes from 46 papers included in systematic review.

Paper ID	Title	Year of Publication	Comparison	Average Shannon's diversity index (H)		Average Chao1 index		Species richness (observed species/ observed OTUs)		Evenness		Alpha diversity		Simpson diversity	
				Control	Variable	Control	Variable	Control	Variable	Control	Variable	Control	Variable	Control	Variable
1	Metagenomic analysis of the uterine bacterial microbiota in healthy and metritic postpartum dairy cows	2011	Healthy and metritis	1.28	1.84	15	51.25								
2	Investigation of postpartum dairy cows' uterine microbial diversity using metagenomic pyrosequencing of the 16S rRNA gene	2012	NA												
3	Characterisation of the bacterial microbiota of the vagina of dairy cows and isolation of pediocin-producing Pediococcus acidilactici	2013	NA												
4	Dynamics of uterine infections with Escherichia coli, Streptococcus uberis and Trueperella pyogenes in post-partum dairy cows and their association with clinical endometritis	2014	NA												
5	Characterization of the vaginal microbiota of ewes and cows reveals a unique microbiota with low levels of lactobacilli and near-neutral pH	2014	General	3.64											
6	Vaginal microbiome characterization of Nellore cattle using metagenomic analysis	2015	Pregnant and non-pregnant	1.79	1.67										
7	Diversity and health status specific fluctuations of intrauterine microbial communities in postpartum dairy cows	2015	Postpartum with different VDS					202							
8	Revisiting bovine pyometra - new insights into the disease using a culture-independent deep sequencing approach	2015	Pyometra		0.97										
9	Uterine microbiota progression from calving until establishment of metritis in dairy cows	2015	Healthy and metritis	2.53	2.2	356	243	443	320						
10	Qualitative analysis of the vaginal microbiota of healthy cattle and cattle with genital-tract disease	2015	Healthy and multiple diseases					15	68						
11	Vaginal microbial communities from synchronized heifers and cows with reproductive disorders	2016	NA												
12	An investigation of the microbiota in uterine flush samples and endometrial biopsies from dairy cows during the first 7 weeks postpartum	2016	Healthy and endometritis	1.7	1.5										
12	An investigation of the microbiota in uterine flush samples and endometrial biopsies from dairy cows during the first 7 weeks postpartum	2016	Healthy and metritis	1.7	1.35										
13	Comparison of vaginal microbial community structure in healthy and endometritis dairy cows by PCR-DGGE and real-time PCR	2016	Healthy and endometritis			1.64	2.27	6.6	11	0.89	0.96				
14	Uterine microbiota and immune parameters associated with fever in dairy cows with metritis	2016	Healthy and metritis	2.55	2.25	580	410								
15	Genetic and functional analysis of the bovine uterine microbiota. Part II: Purulent vaginal discharge versus healthy cows	2017	NA												
16	Genetic and functional analysis of the bovine uterine microbiota. Part I: Metritis versus healthy cows	2017	Healthy and metritis									18	57		
17	Vaginal and Uterine Bacterial Communities in Postpartum Lactating Cows	2017	Vagina and Uterus	7.34	5.83	882	608	838	495						
18	Characterization and identification of microbial communities in bovine necrotic vulvovaginitis	2017	BNVV and BVV	0.8	0.75			83	212	2.36	2.8			0.77	0.73
19	Hot topic: 16S rRNA gene sequencing reveals the microbiome of the virgin and pregnant bovine uterus	2017	Environment and pregnant	5.5	4	1000	200								
20	Dynamics of the microbiota found in the vaginas of dairy cows during the transition period: associations with uterine diseases and reproductive outcome	2017	Healthy and endometritis	7.28	7.28										

Paper ID	Title	Year of Publication	Comparison	Average Shannon's diversity index (H)		Average Chao1 index		Species richness (observed species/ observed OTUs)		Evenness		Alpha diversity		Simpson diversity	
				Control	Variable	Control	Variable	Control	Variable	Control	Variable	Control	Variable	Control	Variable
20	Dynamics of the microbiota found in the vaginas of dairy cows during the transition period: associations with uterine diseases and reproductive outcome	2017	Healthy and fever	7.25	7.08										
20	Dynamics of the microbiota found in the vaginas of dairy cows during the transition period: associations with uterine diseases and reproductive outcome	2017	Healthy and metritis	7.3	7.18										
20	Dynamics of the microbiota found in the vaginas of dairy cows during the transition period: associations with uterine diseases and reproductive outcome	2017	Healthy and retained placenta	7.15	7.55										
21	Blood as a route of transmission of uterine pathogens from the gut to the uterus in cows	2017	Uterus and blood	2.1	0.9	650	425								
21	Blood as a route of transmission of uterine pathogens from the gut to the uterus in cows	2017	Uterus and stool	2.1	4	650	575								
22	Unravelling vaginal microbial genetic diversity and abundance between Holstein and Fleckvieh cattle	2017	Holstein and Fleckvieh	7.464	8.16833333	1577.5725	1721.7485							0.9615	0.9715
23	Microbial communities and inflammatory response in the endometrium differ between normal and metritic dairy cows at 5-10 days post-partum	2018	Healthy and metritis	1.5	1.5										
24	Biogeographical Differences in the Influence of Maternal Microbial Sources on the Early Successional Development of the Bovine Neonatal Gastrointestinal tract	2018	Maternal and calf			213		152							
25	Uterine microbiota of dairy cows with clinical and subclinical endometritis	2018	Healthy and endometritis	3.5	3.5										
26	Shift of uterine microbiota associated with antibiotic treatment and cure of metritis in dairy cows	2018	Untreated and AMP	1.892	2.104										
26	Shift of uterine microbiota associated with antibiotic treatment and cure of metritis in dairy cows	2018	Untreated and CEF	1.892	1.981										
27	Characterization of the cervical bacterial community in dairy cows with metritis and during different physiological phases	2018	Healthy and metritis	7.4	2										
28	A common vaginal microbiota composition among breeds of Bos taurus indicus (Gyr and Nellore)	2019	NA												
29	Delayed differentiation of vaginal and uterine microbiomes in dairy cows developing postpartum endometritis	2019	Healthy and endometritis	3.46	2.38	1657.6	654.1								
30	Uterine and vaginal bacterial community diversity prior to artificial insemination between pregnant and nonpregnant postpartum cows	2019	Pregnant and non-pregnant	7.65	7.65										
31	Microbiota of newborn calves and their mothers reveals possible transfer routes for newborn calves' gastrointestinal microbiota	2019	calf faeces												
32	Effect of pegbovigrastim administration on the microbiome found in the vagina of cows postpartum	2019	Control and treatment	6	5.97	1733	1836								
32	Effect of pegbovigrastim administration on the microbiome found in the vagina of cows postpartum	2019	Healthy and endometritis	6.17	5.43	1933	1633								
32	Effect of pegbovigrastim administration on the microbiome found in the vagina of cows postpartum	2019	Healthy and metritis	6.1	5.5	1850	1700								
33	The relationship between uterine, fecal, bedding, and airborne dust microbiota from dairy cows and their environment: a pilot study	2019	NA												
34	Concurrent and long-term associations between the endometrial microbiota and endometrial transcriptome in postpartum dairy cows	2019	NA												
35	The Bos taurus maternal microbiome: role in determining the progeny early-life upper respiratory tract microbiome and health	2019	Vagina and calf URT	5.8	5.6	2000	2900								
35	The Bos taurus maternal microbiome: role in determining the progeny early-life upper respiratory tract microbiome and health	2019	Vaginal and calf feces	5.8	6.2	2000	1250								
36	The vaginal and fecal microbiomes are related to pregnancy status in beef heifers	2019	Pregnant and non-pregnant	6	6										

Paper ID	Title	Year of Publication	Comparison	Average Shannon's diversity index (H)		Average Chao1 index		Species richness (observed species/ observed OTUs)		Evenness		Alpha diversity		Simpson diversity	
				Control	Variable	Control	Variable	Control	Variable	Control	Variable	Control	Variable	Control	Variable
37	Viral metagenomics reveals significant viruses in the genital tract of apparently healthy dairy cows	2019	NA												
38	Characterization of vaginal microbiota associated with pregnancy outcomes of artificial insemination in dairy cows	2020	Pregnant and non-pregnant	6.5	7.17										
39	Vaginal Microbiota Changes During Estrous Cycle in Dairy Heifers	2020	Follicular and luteus												
40	Study of vaginal microbiota of Holstein cows submitted to an estrus synchronization protocol with the use of intravaginal progesterone device	2020	Control and intravaginal progesterone device												
41	Composition and diversity of the preputial microbiota in healthy bulls	2020	General	2.17											
42	Vaginal bacterial community composition and concentrations of estradiol at the time of artificial insemination in Brangus heifers	2020	Pregnant and non-pregnant	2.18	2.22										
43	Recombinant protein subunit vaccine reduces puerperal metritis incidence and modulates the genital tract microbiome	2020	Unvaccinated and vaccinated	5	5.17										
44	Effect of chitosan microparticles on the uterine microbiome of dairy cows with metritis	2020	Untreated and CM	2.04	2.06										
45	Dynamics of uterine microbiota in postpartum dairy cows with clinical or subclinical endometritis	2020	Healthy and endometritis	3.4	2.9										
46	Determination of uterine bacterial community in postpartum dairy cows with metritis based on 16S rDNA sequencing	2020	Healthy and metritis	3.03	2.05										
